# Prenatal smoking exposure and neuropsychiatric comorbidity of ADHD: a finnish nationwide population-based cohort study

**DOI:** 10.1186/s12888-016-1007-2

**Published:** 2016-08-31

**Authors:** Petteri Joelsson, Roshan Chudal, Ardesheer Talati, Auli Suominen, Alan S. Brown, Andre Sourander

**Affiliations:** 1 Department of Child Psychiatry, University of Turku, Lemminkäisenkatu 3, Lastenpsykiatrian tutkimuskeskus, 20014 Turku, Finland; 2 Department of Psychiatry, Columbia University College of Physicians and Surgeons, New York, NY USA; 3 Division of Epidemiology, New York State Psychiatric Institute, New York, NY USA; 4 Department of Epidemiology, Columbia University Mailman School of Public Health, New York, NY USA; 5 Department of Child Psychiatry, Turku University Hospital, Turku, Finland

**Keywords:** ADHD, Risk factor, Smoking, Tobacco, Conduct disorder, Oppositional defiant disorder

## Abstract

**Background:**

Prenatal smoking exposure has been associated with attention-deficit/hyperactivity disorder (ADHD). ADHD is commonly associated with a wide spectrum of psychiatric comorbidity. The association between smoking and neuropsychiatric comorbidity of ADHD has remained understudied. The aim of this study is to examine the association between prenatal exposure to maternal smoking and offspring ADHD, and test whether the smoking-ADHD associations are stronger when ADHD is accompanied by other lifetime neuropsychiatric comorbidities.

**Methods:**

The study is based on a nested case-control design and includes all Finnish singletons born between 1991 and 2005 and diagnosed with ADHD by 2011 (*n* = 10,132), matched with four controls (*n* = 38,811) on date of birth, sex and residence in Finland.

**Results:**

The risk for ADHD with or without comorbidity was significantly increased among offspring exposed to maternal smoking on adjusting for potential confounders (OR = 1.75, CI 95 % = 1.65–1.86). Compared to the only ADHD cases, subjects with comorbid conduct disorder or oppositional defiant disorder had a significantly stronger association with smoking exposure (OR = 1.80, CI 95 % = 1.55–2.11).

**Conclusions:**

*Prenatal smoking represents an important risk factor for the ADHD comorbid with CD*/*ODD*. Further research on the association between prenatal smoking exposure and neuropsychiatric comorbidity of ADHD is needed considering the increased risk among these subjects of an overall poor health outcome as compared to only ADHD. In particular, studies *utilizing biomarkers or* including subjects with neuropsychiatric conditions with and without comorbid ADHD are needed.

**Electronic supplementary material:**

The online version of this article (doi:10.1186/s12888-016-1007-2) contains supplementary material, which is available to authorized users.

## Background

Attention-deficit/hyperactivity disorder (ADHD) is estimated to have a worldwide prevalence of 5 % [1]. The disorder is characterized by impaired attention, hyperactive and impulsive symptoms with an onset at childhood [2, 3]. ADHD is commonly associated with a wide spectrum of psychiatric comorbidity. *Among ADHD cases*, *comorbid diagnosis of autism spectrum disorders* (*ASD*) *are found in 10*–*33* % [4, 5], *conduct disorder* (*CD*) *in 16*–*18* % [6, 7], *learning and coordination disorders in 15*–*46* % [4, 6, 7] *and mental retardation in 4*–*8* % [4, 6].

Both genetic and environmental risk factors contribute to the development of ADHD [8]. Maternal smoking during pregnancy has been consistently associated with an increased risk of ADHD in the offspring [9, 10]. Recent epidemiological studies, however, suggest that the association between maternal smoking during pregnancy and ADHD may be largely attributable to familial confounders, such as genetic traits and social factors [11, 12]. The possibility of the teratogenic effects of smoking as a cause of ADHD should be considered, because some animal models suggest in utero nicotine exposure to be associated with neuronal cell death [13]. Smoking during pregnancy has been represented to associate with gestational hypoxia [14] and gestational hypoxia has been suggested to contribute to ADHD-like symptoms [15]. Maternal smoking during pregnancy has also been associated with increased offspring risk for several psychiatric conditions, such as pervasive developmental disorders [16], conduct disorders [17] and depression [18].

Psychiatric comorbidity complicates the assessment of ADHD [19] and might cause increasing occupational and educational disability [20]. There are few studies that have examined the association between maternal smoking during pregnancy and ADHD associated with psychiatric comorbidity, focusing mainly on conduct disorders. The results of a cross-sectional Australian study indicated that maternal smoking during pregnancy doubled the ADHD subjects’ risk of being diagnosed with oppositional defiant behavior (ODD). The study was based on parental questionnaires and thus, recall bias may have contributed to the association [21]. A Norwegian study showed that smoking during pregnancy predicted increased risk for comorbid ADHD and ODD [22]. However, those findings were limited by the lack of adjustment for parental psychiatric history. Given evidence showing an increasing burden of psychiatric diseases among smokers in more recent decades [23], the potential for confounding by this covariate could be especially problematic. In this study, we examined whether maternal smoking during pregnancy is associated with varying risk among ADHD cases with different comorbid neuropsychiatric conditions, adjusting for parental psychiatric history and other potential confounders. Specifically, we tested the hypothesis that prenatal smoking exposure has a greater risk among ADHD cases with comorbid psychiatric diagnoses than cases without comorbidity. We hypothesized that comorbid ADHD and CD/ODD shows the strongest association with prenatal exposure to smoking among ADHD with all co-morbid conditions. The hypotheses were based on previous study findings showing that gestational smoking exposure is associated with overall psychiatric morbidity.

## Methods

This is a nationwide register study based on a nested case–control design. The sampling frame included all 900 603 liveborn children in Finland between January 1, 1991 and December 31, 2005. We utilized data from record linkages of three national registers: the Finnish Hospital Discharge Register (FHDR), Finnish Medical Birth Register (FMBR) and Finnish Central Population Register. The cases and controls were identified from the FHDR and the Finnish Central Population Register, respectively. Linkage was achieved by personal identity codes (PIC), which are assigned to all Finnish citizens and residents by the Central Population Register since 1971.

### National register information

The FMBR, founded in 1987 contains standardized data on the perinatal period for all live births, and stillbirths with birth weight of at least 500 g or gestational age of at least 22 weeks in Finland. The FHDR includes medical diagnoses made in the Finnish public health care system. The register covers all inpatient wards, in both somatic and psychiatric hospitals, local health centres, military wards, prison hospitals, and private hospitals. Since 1998, it has also covered outpatient care in public specialized hospitals. In Finland, diagnostic classification is based on the International Classification of Diseases (ICD); ICD-8 (WHO 1967) from 1969 to 1986, ICD-9 (WHO 1977) from 1987 to 1995 and ICD-10 (WHO 1992) from 1996 onwards. This register was used in order to identify cases of ADHD and of co-morbid conditions and parental psychiatric history. The Finnish Central Population Register is a digital national archive containing basic information on Finnish citizens and permanent residents. It is maintained by the Population Register Centre and local register offices. Individual data recorded in the system includes name, personal identity code (PIC), address, citizenship, native language, family relations, date of birth, date of death, emigration/immigration, and other information. The linkage between the registers was made using the PIC, which is unchanged throughout the lifespan.

### Study subjects

The cases consisted of all Finnish singletons (citizens or permanent residents) born between January 1, 1991 and December 31, 2005 and who were diagnosed with ADHD (ICD-10: F90.X or ICD-9: 314.X) by December 31, 2011 while treated in Finnish public special health care after the age of 2 years. Among 10,409 total cases, information on maternal smoking during pregnancy was available for 10,132, which represent the cases for this study.

For every case, we identified four controls matched by sex, date of birth (+/- 30 days) and place of birth, excluding any diagnosis of ADHD or conduct disorders resulting in 40,141 subjects. Among them, maternal smoking status during pregnancy was available on 38,811 subjects who were eventually included in this study. Study subjects with severe or profound mental retardation were excluded from both the case and control groups because it is difficult to assess the symptoms of attentional and hyperkinetic disorders among these subjects. *The controls with CD*/*ODD were not included in the dataset because the clinical features of CD*/*ODD and ADHD overlap and these disorders are difficult to differentiate from one another* [24]. *The case and control identifications were based on hospital diagnoses*, *and structured diagnostic interviews were not conducted to fully confirm whether the control group is entirely free of ADHD. In order to reduce this possibility*, *these controls were not included*. The cases were identified from the FHDR and the controls were identified from the Finnish Central Population Register according to above-named criteria.

### Maternal smoking during pregnancy

Information on maternal smoking during pregnancy was obtained from the FMBR. These were ascertained by maternity clinic nurses during routine prenatal visits during the second trimester of pregnancy and documented in health records. Maternal smoking during pregnancy was classified as: non-smokers, smoking only during the first trimester and smoking after first trimester.

### Covariates

The covariates used in the analyses were: maternal and paternal psychiatric history, maternal history of any substance use, maternal and paternal age at birth of offspring, maternal and paternal immigrant status, maternal socioeconomic status (SES), birth weight for gestational age, Apgar scores at 1 min, number of previous births *and gestational age*. Covariates were classified as represented in Tables 1 and 2. Data on maternal and paternal age and immigrant status were obtained from the Finnish Central Population Register. Maternal SES, maternal marital status, birth weight for gestational age, Apgar scores at 1 min and *gestational age* were obtained from the FMBR.

**Table 1:** Potential confounding factors in relation to maternal smoking during pregnancy in controls

		No smoking	Smoking only in first trimester	Smoking after first trimester	*p*	*χ* ^2^
Maternal age at birth	≤19 years	529 (1.7 %)	48 (7.9 %)	322 (6.4 %)	*p* < 0.001	647.55
	20–29 years	15,660 (50.1 %)	365 (59.8 %)	2836 (56.2 %)		
	30–39 years	14,195 (45.4 %)	186 (30.5 %)	1762 (34.9 %)		
	≥40 years	886 (2.8 %)	11 (1.8 %)	123 (2.4 %)		
Paternal age at birth^a^	≤19 years	106 (0.3 %)	11 (1.9 %)	95 (1.9 %)	*p* < 0.001	413.72
	20–29 years	11,414 (36.8 %)	297 (49.9 %)	2255 (45.9 %)		
	30–39 years	16,485 (53.1 %)	247 (41.5 %)	2160 (44.0 %)		
	≥40 years	3052 (9.8 %)	40 (6.7 %)	404 (8.2 %)		
History of maternal psychopathology	Yes	3321 (10.6 %)	92 (15.1 %)	927 (18.4 %)	*p* < 0.001	258.83
	No	27,949 (89.4 %)	518 (84.9 %)	4116 (81.6 %)		
History of maternal substance abuse	Yes	357 (1.1 %)	22 (3.6 %)	368 (7.3 %)	*p* < 0.001	837.97
	No	30,913 (98.9 %)	588 (96.4 %)	4675 (92.7 %)		
History of paternal psychopathology^a^	Yes	3387 (10.9 %)	109 (18.3 %)	1101 (22.4 %)	*p* < 0.001	528.64
	No	27,670 (89.1 %)	486 (81.7 %)	3813 (77.6 %)		
Previous births	0	12,557 (40.2 %)	381 (62.5 %)	2139 (42.4 %)	*p* < 0.001	129.23
	≥1	18,713 (59.8 %)	229 (37.5 %)	2904 (57.6 %)		
Weight for gestational age^b^	<-2 SD	716 (2.3 %)	16 (2.6 %)	285 (5.7 %)	*p* < 0.001	219.66
	-2 to +2 SD	29,330 (94.1 %)	571 (93.9 %)	4641 (92.4 %)		
	≥ + 2 SD	1141 (3.7 %)	21 (3.5 %)	95 (1.9 %)		
Maternal SES	Upper white collar	5325 (17.0 %)	40 (6.6 %)	263 (5.2 %)	*p* < 0.001	1179.59
	Lower white collar	14,404 (46.1 %)	234 (38.4 %)	1890 (37.5 %)		
	Blue collar	4809 (15.4 %)	161 (26.4 %)	1552 (30.8 %)		
	Others	4663 (14.9 %)	129 (21.2 %)	901 (17.9 %)		
	Missing	2069 (6.6 %)	46 (7.5 %)	437 (8.7 %)		
Maternal immigrant status	Not born in Finland	491 (1.6 %)	13 (2.1 %)	38 (0.8 %)	*p* < 0.001	21.91
	Born in Finland	30,779 (98.4 %)	597 (97.9 %)	5005 (99.2 %		
Paternal immigrant status^a^	Not born in Finland	557 (1.8 %)	9 (1.5 %)	85 (1.7 %)	*p* < 0.841	0.35
	Born in Finland	30,500 (98.2 %)	586 (98.5 %)	4829 (98.3 %)		
Apgar score at 1 min^c^	<6	1151 (3.7 %)	30 (4.9 %)	195 (3.9 %)	*p* < 0.229	2.95
	7–10	30,086 (96.3 %)	577 (95.1 %)	4841 (96.1 %)		
*Gestational age* ^d^	≤*32 weeks*	*195* (*0.6* %)	*3* (*0.5* %)	*47* (*1.0* %)	*p* < *0.029*	*14.11*
	*33*–*36 weeks*	*1171* (*3.8* %)	*19* (*3.1* %)	*218* (*4.3* %)		
	*37*–*41 weeks*	*28*,*407* (*91.0* %)	*553* (*90.8* %)	*4546* (*90.5* %)		
	≥*42 weeks*	*1431* (*4.6* %)	*34* (*5.6* %)	*212* (*4.2* %)		

^a^data missing: 357 subjects

^b^data missing: 145 subjects

^c^data missing 43 subjects

^d^data missing 87 subjects

**Table 2:** Potential confounding factors in relation to case-control status

		Cases	Controls	*p*	*χ* ^2^
Maternal age at birth	≤19 years	672 (6.7 %)	899 (2.4 %)	*p* < 0.001	603.69
	20–29 years	5711 (56.5 %)	18,861 (51.1 %)		
	30–39 years	3475 (34.4 %)	16,143 (43.7 %)		
	≥40 years	254 (2.5 %)	1020 (2.8 %)		
Paternal age at birth^a^	≤19 years	211 (2.2 %)	212 (0.6 %)	*p* < 0.001	330.41
	20–29 years	4322 (44.2 %)	13,966 (38.2 %)		
	30–39 years	4358 (44.5 %)	18,892 (51.7 %)		
	≥40 years	896 (9.2 %)	3496 (9.6 %)		
History of maternal psychopathology	Yes	2610 (25.8 %)	4340 (11.8 %)	*p* < 0.001	1154.75
	No	7502 (74.2 %)	32,583 (88.3 %)		
History of maternal substance abuse	Yes	685 (6.8 %)	747 (2.0 %)	*p* < 0.001	525.72
	No	9427 (93.2 %)	36,176 (98.0 %)		
History of paternal psychopathology^a^	Yes	2382 (24.3 %)	4597 (12.6 %)	*p* < 0.001	779.19
	No	7405 (75.7 %)	31,969 (87.4 %)		
Previous births	0	4683 (46.3 %)	15,077 (40.8 %)	*p* < 0.001	98.03
	≥1	5429 (53.7 %)	21,846 (59.2 %)		
Weight for gestational age^b^	<-2 SD	569 (5.6 %)	1017 (2.8 %)	*p* < 0.001	189.09
	-2 to +2 SD	9167 (91.0 %)	34,542 (93.8 %)		
	≥ + 2 SD	338 (3.4 %)	1257 (3.4 %)		
Maternal SES	Upper white collar	861 (8.5 %)	5628 (17.7 %)	*p* < 0.001	593.56
	Lower white collar	3986 (39.4 %)	6522 (15.2 %)		
	Blue collar	2439 (24.1 %)	16,528 (44.8 %)		
	Others	1959 (19.4 %)	5693 (15.4 %)		
	Missing	867 (8.6 %)	2552 (6.9 %)		
Maternal immigrant status	Not born in Finland	223 (2.2 %)	542 (1.5 %)	*p* < 0.001	26.04
	Born in Finland	9889 (97.8 %)	36,381 (98.5 %)		
Paternal immigrant status^a^	Not born in Finland	368 (3.8 %)	651 (1.8 %)	*p* < 0.001	136.41
	Born in Finland	9419 (96.2 %)	35,915 (98.2 %)		
Apgar score at 1 min^c^	<6	537 (5.3 %)	1376 (3.7 %)	*p* < 0.001	48.65
	7–10	9565 (94.7 %)	35,504 (96.3 %)		
*Gestational age*	≤*32 weeks*	*224* (*2.2* %)	*245* (*0.7* %)	*p* < *0.001*	*219.24*
	*33*–*36 weeks*	*564* (*5.6* %)	*1408* (*3.8* %)		
	*37*–*41 weeks*	*8833* (*87.6* %)	*33*,*506* (*91.0* %)		
	≥*42 weeks*	*460* (*4.6* %)	*1677* (*4.5* %)		

^a^data missing 682 subjects

^b^data missing: 107 subjects

^c^data missing 53 subjects

A father was defined as having a “psychiatric history” if he had been diagnosed with any mental health disorder based on the classification of ICD-8 from year 1969 to 1986 (291-308), ICD-9 from year 1987 to 1995 (291-316) or ICD-10 from year 1996 onwards (F10-99, excluding mental retardation F70-79). Maternal psychopathology was defined correspondingly with the exception of separating “history of substance use history” and “any other psychiatric history” as independent covariates. These were separated because maternal diagnosis of substance use might be an indicator of teratogenic risk factors and thus it differs from paternal substance use diagnosis and other maternal diagnoses. A mother was defined as having a “history of substance use disorder” if she had been diagnosed accordingly based on the classification of ICD-8 from year 1969 to 1986 (291, 303, 304), ICD-9 from year 1987 to 1995 (291, 292, 303,304,305) or ICD-10 from year 1996 onwards (F10-19). Data on parental psychiatric diagnoses were obtained from the FHDR.

### Psychiatric Comorbidity

The lifetime comorbid psychiatric diagnoses of the subjects were obtained from the FHDR according to ICD-10 (F10-F99) similar to that used in the case identification 2011. Only those psychiatric conditions with onset typically during early childhood and with high rates of comorbidity with ADHD were included in the study. These conditions were mental retardation (F70–F79), pervasive developmental disorders, including autism spectrum disorders (ASD) (F84); Tourette syndrome (F95.2); conduct disorders, including oppositional defiant disorder (CD/ODD) (F90.1, F91, F92) and learning and coordination disorders (F80–F83). The corresponding comorbid psychiatric conditions were classified into five groups.

*Seventy-six percentof the ADHD cases had at least one comorbid diagnosis. Learning and coordination disorder was the most common comorbid diagnostic group* (*48.1* %). *The other most common comorbid diagnoses were*: *CD*/*ODD* (*28.1* %), *anxiety disorders* (*13.6* %), *autism spectrum disorders* (*11.9* %), *depression* (*8.9* %,) *and mental retardation* (*3.7* %). *The psychiatric comorbid patterns of the sample have been reported recently in more detail* [25]. *These findings were in general consistent with previous studies* [5]. *The overlap between the observed comorbidities is shown in* Additional file 1: Table S1.

*Because these comorbidities showed significant overlap each case was assigned to only one comorbid group in a hierarchical manner in the following order*: *first mental retardation*; *second ASD*; *third Tourette syndrome*; *fourth CD*/*ODD and fifth learning and coordination disorders*. For example, if a case was diagnosed with both mild mental retardation and Tourette syndrome, she/he would be assigned to the group of mental retardation. If the case had none of the previous conditions, he/she would be classified into group six, “ADHD without comorbidities”. As listed in Table 4, the hierarchy was based on the chronicity, severity and impairment of functional abilities in each disorder. For example, mental retardation and autism are consistently severe and lifelong conditions which impair daily activities.

### Statistical analyses

Multivariable analyses were conducted initially to test the significance of association between covariates and maternal smoking among controls as well as between covariates and ADHD diagnosis using Pearson’s *χ*^2^ -test. The primary outcome variable was an ADHD diagnosis (no/yes). Conditional logistic regression models were used to examine the association between maternal smoking and ADHD with odds ratios (OR) and 95 % confidence intervals (CI).

The unadjusted model was used to evaluate the association between maternal smoking and the outcome in the entire sample. Maternal smoking was classified first as a dichotomous variable and then as a categorical variable by timing during gestation (no smoking /during first trimester only/after first trimester). The multivariable logistic regression model was used to evaluate the associations adjusting for covariates. In this comparison the reference group were the controls. Statistical significance (2-tailed) was judged at *p* <0.05.

Subsequently, we compared the relationships between maternal smoking and odds for ADHD alone and with each comorbid condition. Finally, we compared the relationships between maternal smoking and each ADHD-comorbid group with the “ADHD without comorbidities”. In the multivariable regression model adjustment was made for covariates with associations between both smoking and the outcome covariates at *p* < 0.1. The interaction term between ADHD with each comorbidity versus “ADHD without comorbidity” was assessed to compare the magnitude of ORs for smoking during pregnancy. In addition, differences in the magnitude of the ORs for ADHD between smoking only during the first trimester and smoking after the first trimester were tested correspondingly.

Statistical analyses were performed with SAS statistical software [26].

## Results

Among the potential covariates, maternal and paternal psychiatric history, maternal substance abuse history, maternal and paternal age at birth, maternal socioeconomic status, weight for gestational age, number of previous births *and gestational age* were associated with both maternal smoking during pregnancy among the controls and ADHD and were included as covariates in the analyses (Tables 1 and 2). *Weight for gestational age and gestational age were not included simultaneously as covariates in analyses because of their potential multicollinearity*.

As shown in Table 3, 30.3 % of the mothers of cases smoked during pregnancy, whereas the corresponding figure was 15.3 % for the mothers of controls. The odds for ADHD in the entire sample was significantly increased among subjects exposed to maternal smoking during pregnancy when adjusted for confounders (OR 1.73, 95 % CI: 1.62–1.84, *p* < 0.001). Among the cases, 2.2 % of mothers smoked only during the first trimester and 28.1 % after the first trimester. Among controls, the corresponding figures were 1.6 % and 13.7 %. There were increased odds for ADHD associated with maternal smoking after first trimester (adjusted OR 1.79, 95 % CI: 1.68–1.92, *p* < 0.001) as well as smoking only during the first trimester (OR 1.24, 95 % CI: 1.03–1.50, *p* < 0.012). There were significant differences in the magnitude of the odds for ADHD between smoking only during the first trimester and smoking after first trimester (*p* < 0.001). The difference in odds for ADHD between smoking during pregnancy and during the first trimester only was statistically significant (*p* < 0.001).

**Table 3:** Associations between ADHD and maternal smoking during pregnancy

	Frequencies		OR (95 % CI)	
Maternal smoking status	Cases *n* (%)	Controls *n* (%)	Unadjusted	Adjusted^a^
Not smoking during pregnancy	7068 (69.7 %)	33,158 (84.7 %)	Reference	Reference
Smoking during pregnancy (Yes/No)	3064 (30.3 %)	5653 (15.3 %)	2.44 (2.32–2.58)	1.75 (1.65–1.86)
Smoking after first trimester	2839 (28.1 %)	5043 (13.7 %)	2.54 (2.41–2.68)	1.81 (1.71–1.93)
Smoking only during first trimester	225 (2.2 %)	610 (1.7 %)	1.66 (1.41–1.94)	1.25 (1.05–1.48)

*OR* odds ratio, *CI* confidence interval

^a^Adjusted for maternal and paternal psychiatric history, maternal substance use history, maternal and paternal age at birth, maternal socioeconomic status, birth weight for gestational age, number of previous births and maternal marital status

The associations between maternal smoking during pregnancy and odds of ADHD with each comorbid condition are shown in the Table 4. Smoking during pregnancy was examined as a binary variable (yes/no) instead of considering specific periods because the number of subjects exposed to smoking only during the first trimester was not sufficient for statistical analysis in each diagnostic group. Smoking during pregnancy was a risk factor for ADHD with each comorbid disorder. The highest risk was seen with comorbid CD/ODD (OR = 2.54 95 % CI 2.24–2.88, *p* < 0.001) and the lowest with learning disorders (OR = 1.51 95 % CI 1.35–1.68, *p* < 0.001). *In addition with other confounders the results were adjusted first with weight for gestational age* (*ADHD without comorbidities OR 1.59*, *95* % *CI 1.43*–*1.77*), *second with gestational age* (*OR 1.61 95* % *CI 1.45*–*1.78*) *and third with neither* (*OR 1.61*, *95* % *CI 1.45*–*1.79*). *The results were similar in all three models for all comorbid categories*.

**Table 4:** Associations between ADHD and maternal smoking during pregnancy among comorbid neuropsychiatric conditions

	Cases exposed to smoking during pregnancy *n* (%)	Controls exposed to smoking during pregnancy *n* (%)	OR (95 % CI) Unadjusted	OR (95 % CI) Adjusted^a^
ADHD + Mental Retardation (*N* = 374)	106 (28.3 %)	173 (12.6 %)	2.79 (2.10–3.72)	1.93 (1.38–2.69)
ADHD + ASD (*n* = 1134)	299 (26.4 %)	593 (14.3 %)	2.14 (1.82–2.51)	1.59 (1.33–1.91)
ADHD + Tourette (*n* = 172)	45 (26.2 %)	89 (14.3 %)	2.06 (1.36–3.13)	1.74 (1.06–2.86)
ADHD + CD/ODD (*n* = 2339)	950 (40.6 %)	1308 (15.4 %)	3.96 (3.55–4.41)	2.54 (2.24–2.88)^b^
ADHD + Learning and coordination disorders (*n* = 2957)	799 (27.0 %)	1726 (16.0 %)	1.98 (1.80–2.19)	1.51 (1.35–1.68)
ADHD without comorbidities (*n* = 3136)	865 (27.6 %)	1764 (15.4 %)	2.11 (1.92–2.32)	1.59 (1.43–1.77)

Reference category in each group were their designated controls

*OR* odds ratio, *CI* confidence interval

^a^Adjusted for:maternal and paternal psychiatric history, maternal substance abuse history, maternal and paternal age at birth, maternal socioeconomic status, birth weight for gestational age, number of previous births and maternal marital status

^b^OR was significantly different from “ADHD without comorbidities”, *p* < 0.001

As shown in Table 5, comparing each ADHD-comorbid group with the “ADHD without comorbidities” the comorbid CD/ODD was the only diagnostic group which had a significantly stronger association with smoking (adjusted OR 1.80 95 % CI 1.55–2.11, *p* < 0.001). The association between smoking and comorbid CD/ODD was significantly stronger compared to the respective associations for each of the other ADHD-comorbid diagnostic groups in unadjusted models. In the adjusted model only the groups of ADHD subjects with comorbid Tourette syndrome and mental retardation did not differ significantly from the group with ADHD and CD/ODD.

**Table 5:** The comparisons of associations between maternal smoking and groups with ADHD and comorbid conditions and with ADHD without co-morbid conditions

	Unadjusted OR (95 % CI)	Adjusted OR^a^ (95 % CI)
ADHD + Mental Retardation (*n* = 374)	1.32 (0.98–1.79)	1.32 (0.96–1.83)
ADHD + ASD (*n* = 1134)	1.01 (0.84–1.22)	0.98 (0.80–1.20)
ADHD + Tourette (*n* = 172)	0.98 (0.64–1.50)	1.06 (0.67–1.68)
ADHD + CD/ODD (*n* = 2339)	1.87 (1.62–2.16)	1.80 (1.55–2.11)
ADHD + Learning and coordination disorders (*n* = 2957)	0.94 (0.82–1.07)	0.96 (0.83–1.11)
ADHD without comorbidities (*n* = 3136)	Reference	Reference

*OR* odds ratio, *CI* confidence interval

^a^Adjusted for: maternal and paternal psychiatric history, maternal substance abuse history, maternal and paternal age at birth, maternal socioeconomic status, birth weight for gestational age and number of previous births

## Discussion

The present study has several findings which add to the evidence of a complex association between exposure to smoking during pregnancy and later ADHD with comorbid conditions. First, prenatal smoking exposure was associated with increased risk of ADHD with comorbid neuropsychiatric disorders. Second, comparing each of the co-morbid groups with the control group, the strongest association was found with ADHD comorbid with CD/ODD. Third, the group with ADHD and CD/ODD was the only group showing a statistically significant association with smoking compared to the group of ADHD without comorbidities. To our knowledge, this is the first study to report these associations in a large nationwide sample.

The association between maternal smoking during pregnancy and, offspring ADHD and, specifically CD/ODD status has several possible explanations. First, exposure to smoking during pregnancy might have direct intrauterine effects mediated by nicotine contributing to risk for ADHD and CD/ODD. Nicotine has been shown to transfer across the placenta to the fetus [27]. Also, nicotine is an agonist of acetylcholine and can in this way interfere with fetal central nervous system maturation. [28]. In animal models nicotine exposure is associated with increased locomotor activity which is considered as an indicator for ADHD [29]. Furthermore, nicotine exposure during pregnancy has been argued to increase risk for later conduct problems [30]. Second, the association of smoking and CD/ODD and ADHD might partially be explained by gene-environment-interaction. Early smoking exposure might increase the risk of comorbid ADHD and CD/ODD particularly among children with a specific genetic vulnerability. For example, children exposed to maternal smoking during pregnancy were given higher hyperactive-impulsive and oppositional scores if they were homozygous for the 10-repeat dopamine transporter gene allele [31]. Third, smoking might be a proxy for risk factors leading to ADHD and CD/ODD with independent mechanisms. The overall tendency to smoke cigarettes correlates with the level of ADHD symptoms [32]. It is possible that among smoking mothers, a significant proportion have sub-clinical ADHD which are not diagnosed in healthcare services and are thus unidentified. Therefore, smoking exposure might be an indicator of genetic risk factors. Simultaneously, smoking is also associated with harsh parenting practices [33] which are associated with disruptive behavior of the offspring [34]. Furthermore, poor parenting skills have been suggested to increase the risk of comorbid psychiatric conditions among children with ADHD who might be especially vulnerable to harsh or non-supportive parenting styles [35].

The association between prenatal smoking exposure and offspring ADHD in general was in line with previous similar studies reporting findings of ORs ranging between 1.5 and 2.4 [9, 10]. The differences of the odds between smoking only during the first trimester and after the first trimester suggest a possibility of a dose-response effect. This hypothesis is supported by a previous finding of a linear correlation between number of cigarettes smoked during pregnancy and severity of attentional problems [36]. The dose-response-effect could be mediated by chronic hypoxia to the fetal brain as animal models have demonstrated association between neonatal hypoxia and ADHD-like behavior [15]. The fetal brain could also be more vulnerable to smoking exposure in the later periods of pregnancy. Alternatively, the ability to quit smoking after the first trimester might be a proxy for unknown protective maternal attributes. As shown in the Table 1, the mothers who smoked after the first trimester had more often a history of psychiatric disorders compared to mothers who ceased smoking. It is unclear whether, for example, bipolar disorder or schizophrenia are more common within mothers smoking after the first trimester, especially given that these parental disorders have been associated with offspring ADHD [37].

The present study is based on a large nationwide sample, and the analyses were adjusted with several confounders. However, there are limitations that need to be considered while interpreting the findings. First, the subjects include only the ADHD cases referred to specialized mental health services and assumingly these represent mainly the patients with the most severe symptoms of ADHD. The healthcare services in Finland are universal and publicly funded; children are exempted from the generally low patient fees. Because of this the number of severe ADHD cases missing due to lack of access to health services is expected to be small. Second, the maternal smoking status was based on a self-report. Previous studies have shown that pregnant mothers under-report their smoking. Actually, between 7 and 22 % of the self-reported non-smokers were smokers according to the blood and urine cotinine levels [38, 39]. Third, there are several potential confounders not available in this study, e.g. paternal smoking, and prenatal alcohol and drug exposure. However, information about maternal substance use diagnosis was included in the analyses. Fourth, we did not have information on children diagnosed only with CD/ODD. Therefore, we are not able to address the question whether the association between smoking and ADHD and CD/ODD could be due to the effects of smoking on CD/ODD independent of ADHD. Indeed, there is evidence of the association between smoking exposure and conduct problems [17]. However, few previous studies have not shown maternal smoking to have an effect on conduct problems when the results have been adjusted with parental psychiatric history and socioeconomic factors [40, 41]. Fifth, controls with CD/ODD were excluded which could strengthen the association between smoking and ADHD comorbid with CD/ODD compared to other diagnostic groups. However, the cumulative incidence of CD/ODD diagnosis in Finland by the age of 14 has recently been reported to be only 1.4 % [42] and, thus, the exclusion is expected to have a modest influence to the results at the most. Furthermore, the association between smoking and ADHD with comorbid CD/ODD was significantly stronger compared to the group without comorbidities. This indicates that our findings cannot be explained only by the exclusion of controls with a CD/ODD diagnosis. *Sixth*, *common internalizing disorders*, *such as anxiety and depression*, *were not included in the analysis. Anxiety and depression may have onset at adolescence or adulthood whereas the included disorders typically have an onset at childhood. Including these disorders in the utilized hierarchical model would have been complicated. However*, *exclusion of internalizing comorbidities is not expected to affect the conclusions drawn from our results*.

## Conclusions

The study supports previous findings of association between smoking during pregnancy and offspring ADHD. In future studies utilization of biological markers of smoking such as cotinine levels would confirm the present results. The children with the combination of ADHD and CD/ODD show more tendency to develop mood disorders [43] and later criminal activity [44] compared to children with ADHD only. The strong association between prenatal smoking exposure and comorbid CD/ODD and ADHD warrants further research considering the higher risk among these subjects of an overall poor health outcome compared to only ADHD. Future studies on ADHD and CD/ODD should examine the association between smoking and these disorders as both independent and comorbid conditions. Our findings add to the mounting evidence that smoking during pregnancy has an adverse effect on the developing fetal brain and is associated with the development of neurodevelopmental disorders. Finally, regardless of the precise pathogenic mechanisms, prenatal smoking represents an important risk factor for the ADHD comorbid with CD.

## Additional file

Additional file 1:
**Table S1.** Overlap between the comorbid diagnostic categories. (DOCX 14 kb)

## Abbreviations

ADHD: Attention-deficit/hyperactivity disorder
ASD: Autism spectrum disorders
CD: Conduct disorder
FHDR: Finnish hospital discharge register
FMBR: Finnish medical birth register
ICD: International classification of diseases
ODD: Oppositional defiant behavior
OR: Odds ratio
PIC: Personal identity code
SES: Socioeconomic status
