# Supplementary material for: Prenatal smoking exposure and neuropsychiatric comorbidity of ADHD: a finnish nationwide population-based cohort study
Source: BMC Psychiatry. 2016 Aug 31;16(1):306. doi: 10.1186/s12888-016-1007-2 (PMC5006583; doi:10.1186/s12888-016-1007-2)
Supplement: Additional file 1: — Table S1. Overlap between the comorbid diagnostic categories. (DOCX 14 kb) [file 12888_2016_1007_MOESM1_ESM.docx]

Additional file 1: Table S1

Overlap between the comorbid diagnostic categories

|  | No additional comorbid diagnoses | One additional comorbid diagnosis | Two additional comorbid diagnoses | Three additional comorbid diagnoses | Four additional comorbid diagnoses |
| --- | --- | --- | --- | --- | --- |
| ADHD+  Mental retardation | 36  (9.6%) | 226  (60.4%) | 98  (26.2%) | 13  (3.5%) | 1  (0.3%) |
| ADHD+ASD | 356  (29.6%) | 577  (47.9%) | 240  (20.0%) | 29  (2.4%) | 1  (0.1%) |
| ADHD+  Tourette | 66  (27.3%) | 99  (40.9%) | 60  (24.8%) | 16  (6.6%) | 1  (0.4%) |
| ADHD+  CD/ODD | 1407  (49.6%) | 1136  (40.1%) | 263  (9.2%) | 29  (1.0%) | 1  (0.0%) |
| ADHD+  Learning and coordination  disorder | 2957  (60.9%) | 1572  (32.4%) | 296  (6.1%) | 29  (0.6%) | 1  (0.0%) |
